# Effects of Fly Ash Particle Size and Chemical Activators on the Hydration of High-Volume Fly Ash Mortars

**DOI:** 10.3390/ma17225485

**Published:** 2024-11-10

**Authors:** Young-Cheol Choi, Byoungsun Park

**Affiliations:** 1 Department of Civil and Environmental Engineering, Gachon University, Seongnam-si 13120, Republic of Korea; zerofe@gachon.ac.kr; 2 Department of Environmental Systems Engineering, Sejong Campus, Korea University, Sejong-si 30019, Republic of Korea

**Keywords:** high volume fly ash, particle size, chemical activator, hydration, pozzolan reaction

## Abstract

This study investigated the effects of the fly ash (FA) particle size and chemical activators on the hydration reactions of high-volume fly ash (HVFA) cement pastes and the mechanical properties of HVFA mortars, with five types of FA with varying proportions of particles smaller than 10 μm prepared to assess the influence of particle size. In addition, the effect of chemical admixtures on the early-age hydration of the HVFA cement paste was evaluated. Quartz powder with the same fineness as that of normal FA was prepared to specifically examine the pozzolanic reaction of FA. The hydration reactions of the HVFA cement pastes were analyzed using isothermal calorimetry to measure the heat of hydration for 72 h. The results showed that an increase in the contents of FA particles with a diameter lower than 10 μm increases specific heat flow. Chemical activators, including Na_2_SO_4_, triethanolamine (TEA), and triisopropanolamine (TIPA), promote early-age hydration of HVFA cement pastes. The mechanical properties of the HVFA mortar were evaluated by measuring its compressive strength at 3 d, 7 d, and 28 d, with the findings revealing that the compressive strength of HVFA mortar improved with an increased proportion of FA particles smaller than 10 μm. Na_2_SO_4_, TEA, and TIPA consistently increased the compressive strength of the HVFA mortars.

## 1. Introduction

Considering the cement industry is a significant contributor to CO_2_ emissions, recent efforts have focused on reducing cement usage to meet the carbon neutrality goals. One common strategy for lowering cement consumption involves substituting a large portion of cement with supplementary cementitious materials (SCMs). Prominent industrial byproducts used as SCMs include ground-granulated blast furnace slag (GGBFS) and fly ash (FA). ASTM C595 allows GGBFS, a byproduct of the steel industry, to replace up to 95% of cement content [1]. In contrast, ASTM C595 permits FA to replace up to 40% of cement. FA is a byproduct of coal-fired power plants and is used as a binder for ingredients of concrete, replacing ordinary Portland cement (OPC). Fly ash helps control the hydration heat of concrete and has the advantage of increasing the long-term strength of concrete. However, the extensive use of FA is often restricted owing to its tendency to significantly lower early strength, unlike GGBFS [2,3]. Due to the low reactivity of FA, an increase in the use of FA causes a decrease in the early-age strength of concrete. Therefore, enhancing the early strength of cementitious materials with high FA replacement is crucial for enabling broader use of FA. So, it is necessary to improve the reactivity of FA, to increase the utilization efficiency of FA.

FA enhances the mechanical performance of cementitious materials via two main mechanisms [4,5,6,7,8]. The first is the filler effect, which accelerates the early hydration of ordinary Portland cement (OPC) [9,10,11]. As FA does not participate in the initial hydration stages, it serves as a nucleation site for hydration products, enhancing the hydration process [12,13]. Additionally, as FA does not react at an early age, it improves the packing density by filling the gaps between cement particles, which helps increase the compressive strength [14]. The second mechanism is the pozzolanic reaction of the FA [15], as shown in Equation (1).

Ca(OH)_2_ + SiO_2_ + H_2_O → C-S-H (1)

The pozzolanic reaction of FA begins only after Ca(OH)_2_ is produced by the hydration of OPC, indicating that it does not occur in the early stages. To facilitate the pozzolanic reaction of FA, accelerating cement hydration to ensure early Ca(OH)_2_ formation is essential. The fineness of the FA affects the pozzolanic reaction [16]. Fine FA has a higher vitreous content compared to coarse FA owing to the greater proportion of particles smaller than 45 μm, particularly those under 10 μm [17]. Extremely fine FA particles are rich in reactive components, particularly active Al_2_O_3_ [17]. Małolepszy and Tkaczewska [17] noted that FA particles in the 0–16 μm range, collected by electrostatic precipitators, contain significantly more active chemical components than standard FA, with differences potentially reaching 1.5 times. This effect is largely due to the greater fineness of the 0–16 μm FA particles but also possibly influenced by differences in the chemical composition and structure of the vitreous phase. Erdogdu and Tucker [18] found that fine-sieved FA enhances mortar strength compared to mortar made with coarser FA. Therefore, utilizing highly fine FA is expected to improve the early strength of high-volume fly ash (HVFA) cement.

To accelerate the pozzolanic reaction of FA, Ca(OH)_2_ must be quickly produced through the hydration of OPC. One effective method to enhance OPC hydration involves using chemical admixtures. An alternative method for improving the early strength of the HVFA cement or concrete involves the use of chemical activators. Triethanolamine (TEA) and triisopropanolamine (TIPA) are often used to accelerate cement hydration and increase early strength [19,20]. TEA promotes C_3_A dissolution in OPC and aids ettringite formation, thereby improving the early-age strength of cementitious materials [21]. Some studies have suggested that TEA facilitates the dissolution of aluminum and ferric ions from FA particles via complexation with Al^3+^ and Fe^3+^, which enhances the pozzolanic reaction of FA [22]. Sandberg and Doncaster [23] found that TIPA accelerates OPC hydration by forming a complex with iron (TIPA-Fe), which induces ferric ion dissolution and supports the formation of hydrated calcium sulfoaluminate in the cement paste matrix [24]. In FA–cement systems, the acceleration of cement hydration by TIPA promotes calcium hydroxide (CH) formation, which in turn enhances the pozzolanic reaction of FA. Although TIPA, similar to TEA, may aid in dissolving FA particles, research on this aspect remains limited.

This study investigated the effects of FA particle size and chemical activators on the hydration reactions of HVFA cement and the mechanical properties of HVFA cement mortar, with five types of FA with varying proportions of particles smaller than 10 μm prepared to investigate the influence of particle size. Chemical admixtures were employed to accelerate the early-age hydration of the HVFA cement. To specifically examine the pozzolanic reaction of FA, quartz powder with the same fineness as the FA was prepared and mixed in the same proportions in weight as the FA for the test specimens. The hydration reactions of the HVFA cement pastes were analyzed using isothermal calorimetry to measure the heat of hydration over a 72 h period. Finally, the mechanical properties of the HVFA mortar were evaluated by measuring its compressive strength at 3 d, 7 d, and 28 d. The compressive strength was measured to investigate the effects of FA particle size and chemical activators on the mechanical performance of HVFA cementitious materials at early ages. In addition, hydration heat was measured using isothermal calorimetry to investigate the reactivity of OPC and FA at early ages. The experimental results led to the identification of strategies for enhancing early-age hydration and improving the compressive strength of HVFA cement.

## 2. Materials and Methods

### 2.1. Materials

In this study, OPC and FA were used as binders, with FA sourced from a Yeongheung coal-fired power plant in South Korea. The chemical compositions of OPC and FA are listed in Table 1. The Bogue phase composition of the OPC consisted of 57.9% C_3_S, 16.0% C_2_S, 6.2% C_3_A, and 9.4% C_4_AF by mass. The density and Blaine fineness of OPC were 3.14 g/cm^3^ and 382 m^2^/kg, respectively. The FA used in this study was classified as Class F under ASTM C 618, with a density of 2.2 g/cm^3^ and a Blaine fineness of 387 m^2^/kg. To study compressive strength development owing to the pozzolanic properties of FA, quartz powder was substituted in the same ratio in weight as FA in the specimens as an inert material during fabrication. This approach followed the method outlined by Tangpagasit et al. [25]. Standard sand presented in ISO 679 was used as the fine aggregate to fabricate the mortar specimens for compressive strength testing.

**Figure 1:**
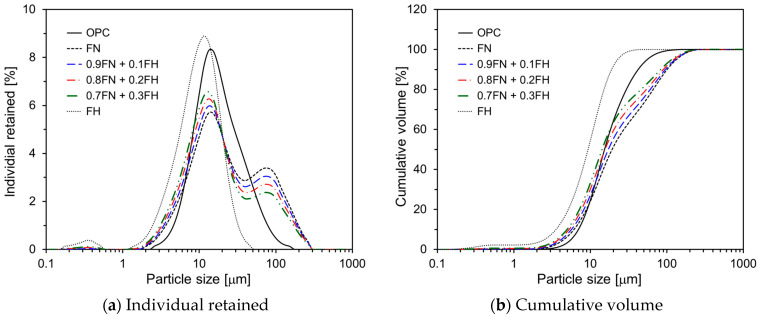
Particle size distributions of OPC and FAs.

Five different types of FA were considered to assess the effect of FA particle size on the hydration and early-age strength of HVFA cement. Initially, standard FA with a median diameter of 19.3 μm (FN) was processed using a ball mill machine to produce fine FA (FH) with a median diameter of 9.2 μm. Subsequently, three additional FA samples with varying particle size distributions were created by replacing 10%, 20%, and 30% of FN by weight with FH. Figure 1 depicts the particle size distributions (PSDs) of OPC and the five types of FA measured by laser diffraction (Beckman Coulter LS 230). While OPC showed a unimodal distribution, FA exhibited a bimodal distribution. Table 2 presents the percentage of particles smaller than 10 μm for each of the five types of FA, as determined from the PSD analysis.

### 2.2. Mixing Proportions and Testing Method

HVFA cement paste and mortar specimens were prepared to examine the effects of the FA particle size and various chemical activators on the hydration and compressive strength. A consistent water-to-binder ratio (0.3) was maintained for all the specimens, with FA comprising 50% of the cement by weight. The specific mixture proportions are listed in Table 3. Five different types of FA, listed in Table 2, were used to investigate the influence of particle size. The chemical activators used were TEA (97%), TIPA (85%), Na_2_SO_4_, sodium carbonate (Na_2_CO_3_), and lithium carbonate (LiCO_3_), with their proportions listed in Table 3. To assess the effect of the pozzolanic reaction of FA on the compressive strength, quartz powder was used as an inert substitute for FA in the same proportion during specimen preparation [26]. The quartz powder was adjusted to have a particle size distribution similar to that of the FN. Mortar specimens were prepared by adding standard sand at 1.7 times the weight of cement to the paste mix, as listed in Table 3.

The hydration heat flow and cumulative heat of the HVFA cement were measured using a TAM-air isothermal conduction calorimeter, which featured eight channels operating in the milliwatt range. Approximately 4 g of paste was placed in a 20 mL glass vial, weighed, and inserted into the calorimeter. The heat flow was recorded automatically for 3 d, with the calorimeter maintained at a steady temperature of 23 ± 2 °C throughout the experiment.

For compressive strength testing of the different mortar mixtures, three mortar prisms were prepared for 3 d, 7 d, and 28 d of curing, following the ISO 679 standards. The mortar specimens were cast in molds measuring 40 × 40 × 160 mm and compacted via vibration. The specimens were initially cured in a controlled environment at 20 ± 1 °C and over 90% relative humidity for the first 24 h. After demolding, they were subjected to water curing at 20 ± 1 °C until the scheduled testing dates.

## 3. Results and Discussion

### 3.1. Hydration Kinetics Characteristics

**Figure 2:**
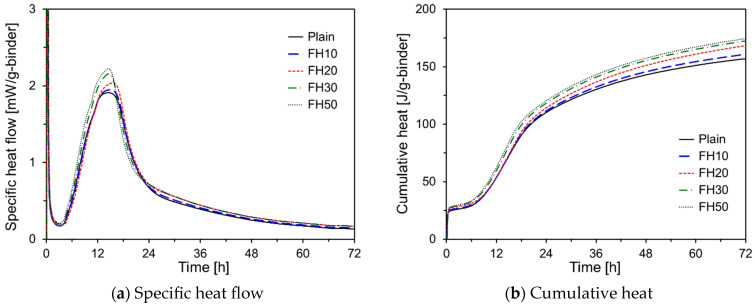
Hydration kinetics of HVFA pastes according to the particle size of FA.

Figure 2 shows the hydration kinetics of the HVFA cement pastes with respect to the FA particle size. The specific heat flow data indicated that the fineness of FA did not affect the induction period. However, as the fineness of the FA increased, the peak during the acceleration period increased notably. Although FA does not actively participate in the early stages of hydration, it enhances OPC hydration through the seeding effect, which provides additional nucleation sites for the hydration products [12,13]. During the acceleration period, calcium silicate hydrates (C-S-H) and CH were formed from C_3_S of OPC, and the higher fineness of FH compared with that of FN likely provided more nucleation sites, thereby accelerating OPC hydration. The trend in the graph after the acceleration period remained consistent regardless of the FH content, suggesting that finer FA primarily accelerated the OPC hydration without significantly influencing the subsequent stages. The cumulative heat also increased with a higher proportion of FH. Without chemical activators, FA typically contributes to hardening through a pozzolanic reaction after 7 d [27]. Thus, the increase in cumulative heat with higher FH content in the HVFA pastes is likely due to enhanced OPC hydration.

**Figure 3:**
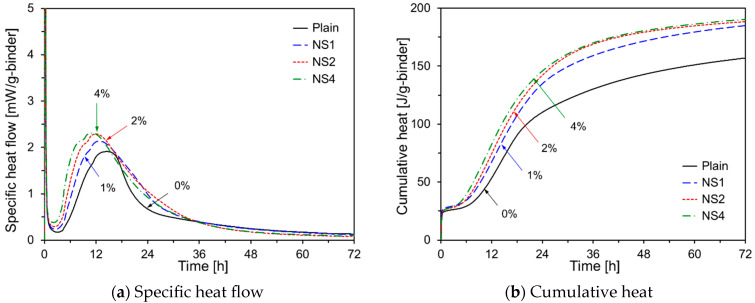
Hydration kinetics of HVFA pastes using Na_2_SO_4_.

Figure 3 depicts the hydration kinetics of the HVFA cement pastes with varying Na_2_SO_4_ contents. As shown in Figure 3a, increasing the Na_2_SO_4_ content shortened the induction period and increased the peak during the acceleration period. Na_2_SO_4_ acted as a catalyst for both OPC hydration and the pozzolanic reaction of FA. The SO_3_^2−^ ions released from Na_2_SO_4_ promoted the early formation of ettringite during the initial stages of OPC hydration, which enhanced the early-age strength. Typically, after the acceleration period, the OPC exhibits a lower peak owing to sulfate depletion. However, in Figure 3a, the sulfate depletion peak is higher than the silicate reaction peak, likely because of the increased concentration of Na_2_SO_4_, which initiates the dissolution of the FA glass phase and accelerates the pozzolanic reaction [28].

**Figure 4:**
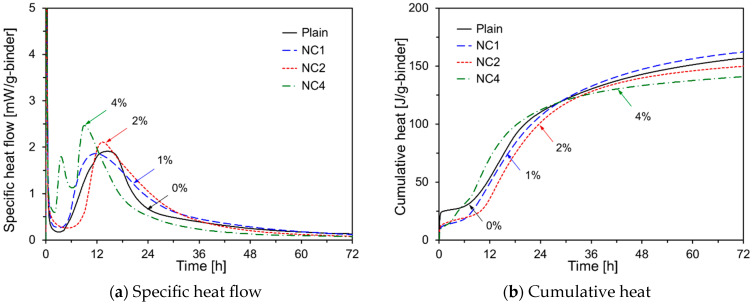
Hydration kinetics of HVFA pastes using Na_2_CO_3_.

Figure 4 shows the hydration kinetics of HVFA cement pastes with different Na_2_CO_3_ contents. As seen in Figure 4a, the early-age hydration kinetics vary depending on the Na_2_CO_3_ content. For NC1, the induction period was similar to that of Plain; however, the secondary peak was reached more quickly, likely due to Na_2_CO_3_ enhancing the dissolution of C_3_S in OPC [29]. The deceleration period following the peak in NC1 is longer. In contrast, NC2 exhibited a longer induction period and a higher secondary peak than Plain, with an extended deceleration period similar to that of NC1. For NC4, an additional third peak was observed. The secondary peak corresponds to the dissolution of C_3_S, whereas the third peak is associated with the dissolution of C_3_A, suggesting that ettringite formation continued even after sulfate depletion [30]. Figure 4b indicates that the cumulative heat for NC1 was lower than that of Plain up to approximately 30 h but increased thereafter. For NC2, the cumulative heat remained consistently lower than that of Plain throughout the 72 h experiment. For NC4, the cumulative heat was higher than that of Plain until approximately 30 h and then decreased, resulting in the lowest overall cumulative heat value. These findings suggest that, although higher concentrations of Na_2_CO_3_ accelerate early-age hydration, they may reduce the overall degree of hydration in the long term.

**Figure 5:**
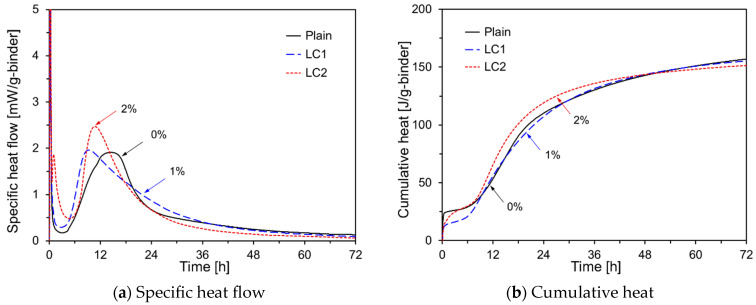
Hydration kinetics of HVFA pastes using LiCO_3_.

Figure 5 shows the hydration kinetics of HVFA cement pastes with different levels of LiCO_3_. As depicted in Figure 5a, LC1 shows a shorter induction period and faster acceleration period than Plain, likely due to LiCO_3_ enhancing the dissolution of C_3_S. In contrast, LC2 exhibited a delayed induction period compared with both Plain and LC1. However, the acceleration period peak was reached more quickly, likely due to the higher concentration of LiCO_3_ promoting further C_3_S dissolution. He et al. studied the effects of LiCO_3_ on cement hydration and found that LiCO_3_ accelerated the OPC hydration and increased the early-age contents of Aft and Ca(OH)_2_ [31]. Figure 5b shows the cumulative heat data. For LC1, the cumulative heat was lower than that of Plain for the first 10 h but became nearly the same thereafter. In contrast, LC2 showed an increase in cumulative heat after 10 h, which decreased after 48 h compared to Plain, thus confirming that as the Li_2_CO_3_ content increased, the initial hydration of OPC increased but decreased after 48 h.

**Figure 6:**
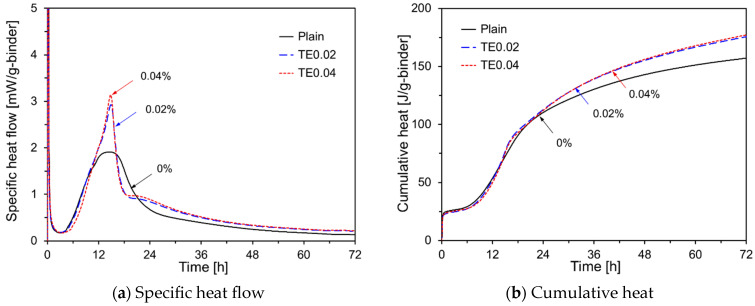
Hydration kinetics of HVFA pastes using TEA.

Figure 6 illustrates the heat evolution of the HVFA cement pastes with added TEA. In Figure 6a, the specific heat flow indicates that TEA-containing specimens exhibit higher second peaks than Plain. Although adding TEA increased the height of the second peak, it did not affect the induction period. In the specimens containing both TIPA and TEA, the specific heat flow decreased rapidly after the second peak, likely because of the greater consumption of C_3_A during the first peak compared to Plain [21]. Figure 6b shows that adding TEA did not influence the specific heat flow within the first 12 h after mixing; however, the cumulative heat levels in the specimens with TEA increased after this period. TEA accelerated the reaction of C_3_A. However, the early-age hydration heat release rate of C_3_A could not be captured because the pastes were mixed outside the isothermal calorimeter. Consequently, changes in specific heat flow owing to TEA addition were not accurately recorded. Therefore, the initial strength improvement of FA50–TEA could only be inferred from the cumulative heat data.

**Figure 7:**
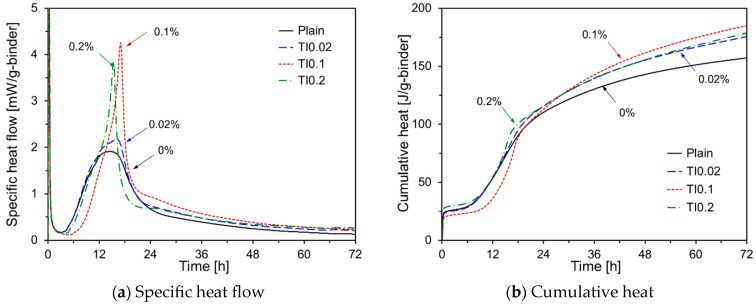
Hydration kinetics of HVFA pastes using TIPA.

Figure 7 illustrates the heat evolution of HVFA cement paste with the addition of TIPA. As shown in Figure 7a, similar to TEA, TIPA increased the intensity of the second peak. For TI0.1, with a TIPA content of 0.1%, the induction period was longer than those of TI0.02 and TI0.2, and it exhibited the highest secondary peak. As the TIPA content increased, the deceleration period became shorter, likely owing to the greater consumption of C_3_A during the first peak. Figure 7b reveals that the cumulative heat for TI0.02 and TI0.2 was similar to that of the Plain for the first 12 h but exceeded it afterward. In the case of TI0.1, the cumulative heat was lower than that of Plain for up to 18 h but became the highest among all samples thereafter, suggesting that a TIPA content of 0.1% is optimal for enhancing the early-age hydration of HVFA.

**Figure 8:**
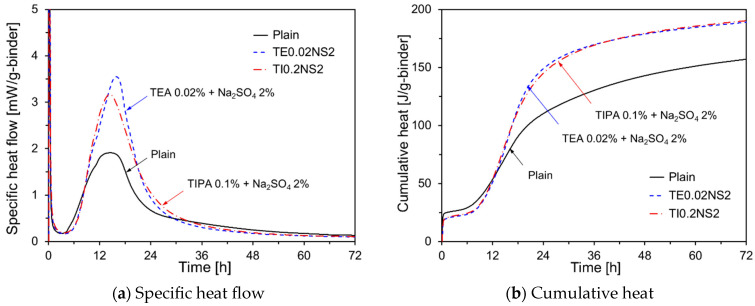
Hydration kinetics of HVFA pastes with combined chemical activators.

The hydration kinetics of HVFA cement were examined using different types and amounts of chemical activators. The findings revealed that Na_2_SO_4_, TEA, and TIPA were the most effective in increasing the cumulative heat over a 72 h period. The hydration kinetics of HVFA cement with mixed activators were studied to determine the optimal combination of chemical activators to enhance the early-age hydration of HVFA cement, with two combinations tested: 2% Na_2_SO_4_ with 0.02% TEA, and 2% Na_2_SO_4_ with 0.1% TIPA. Figure 8 shows the heat evolution of HVFA cement pastes with these combined chemical activators. As illustrated in Figure 8a, both combinations resulted in a higher secondary peak compared with Plain, with a deceleration period similar to that of Plain. When TIPA or TEA was used alone as chemical activators, the rapid dissolution of C_3_A during the first peak led to an increase in aluminate, causing ettringite to quickly convert into monosulfate, which shortened the deceleration period. However, with the addition of Na_2_SO_4_, the SO_4_^2−^ ions stabilized ettringite beyond the secondary peak, preventing its premature conversion [32]. Figure 8b shows that both combinations resulted in similar cumulative heat levels.

### 3.2. Compressive Strength

**Figure 9:**
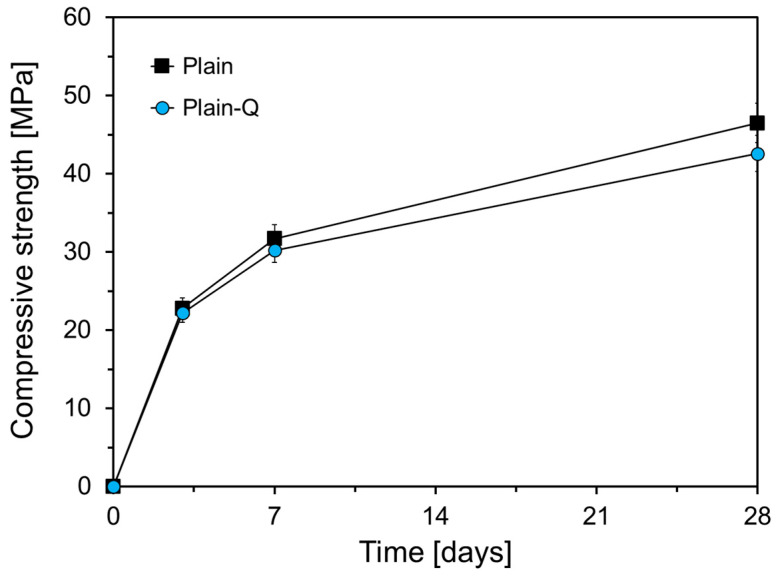
Strength developments of Plan and Plain-Q.

Figure 9 shows the compressive strength results for Plain and Plain-Q. “Plain-Q” refers to specimens in which the FA used in Plain was replaced with inert quartz powder that does not undergo a pozzolanic reaction. Quartz powder was selected to match the particle size distribution of the FA. The compressive strength of Plain-Q was measured to assess the influence of FA’s pozzolanic reaction of FA on strength development over time. As shown in Figure 9, Plain-Q exhibits a lower compressive strength than Plain at all measured ages. Specifically, the compressive strengths of Plain-Q at 3 d, 7 d, and 28 d were 97.4%, 95.3%, and 91.6% of those of Plain, respectively. The compressive strength difference between Plain and Plain-Q increased with age, likely due to the pozzolanic reaction of the FA. During OPC hydration, Ca(OH)_2_ was produced, and FA reacted with Ca(OH)_2_ to form C-S-H [15]. This pozzolanic reaction typically begins after the OPC hydration has advanced, generally starting at approximately 7 d [27]. Thus, the increasing strength difference over time can be attributed to the continued pozzolanic reaction of FA.

**Figure 10:**
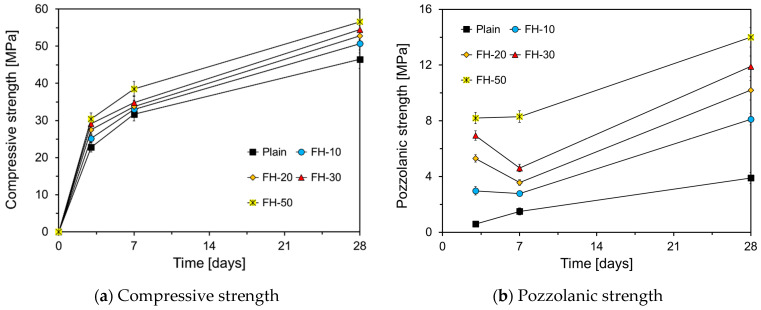
Compressive strength of HVFA mortar according to the particle size of FA.

Figure 10 illustrates the compressive and pozzolanic strengths of HVFA cement mortar. As depicted in Figure 10a, the compressive strength of the specimens increased in the order of FH-50, FH-30, FH-20, FH-10, and Plain up to 28 d, with FH-50 exhibiting the highest compressive strength. The differences in compressive strength between FH-50 and Plain were 7.6 MPa, 6.8 MPa, and 10.1 MPa at 3 d, 7 d, and 28 d, representing gains of 33.3%, 21.5%, and 21.7%, respectively, suggesting that using finely ground FA can significantly enhance the early strength of the HVFA cement mortar. The pozzolanic reaction of FA typically begins at approximately 7 d [27]; therefore, the differences in compressive strength observed for up to 7 d are primarily due to the filling effect of the FA. The differences at 28 d were likely due to the combined effects of both the filling effect and the pozzolanic reaction. Finer FA provides a greater filling effect, resulting in higher compressive strength, and its increased pozzolanic reactivity contributes to an even greater strength at 28 d. Figure 10b summarizes the pozzolanic strength of FA, calculated as the difference between the compressive strength of the specimens shown in Figure 10a and that of Plain-Q specimens shown in Figure 9. Since quartz powder is an inert material and does not contribute to strength development, the compressive strength of Plain-Q is developed only by the hydration of OPC. Therefore, the difference in strength between Plain-Q and FA-added specimens can be considered to be the contribution of the pozzolanic reaction of FA. In this study, the pozzolanic reaction was evaluated using the difference in compressive strength between Plain-Q and HVFA. The pozzolanic strength was influenced by two factors: the filling effect of the FA and the pozzolanic reaction. The graph reveals that the pozzolanic strength increased with FA fineness, regardless of the curing age. For instance, at 3 d, even FH-10, with only 10% of FN replaced, showed a 3 MPa increase in pozzolanic strength, a significant improvement compared to the 0.6 MPa increase observed in Plain, thus highlighting the significant impact of the FA particle size on both the filling effect and the pozzolanic reaction. These results are consistent with the findings of Lee et al. [33], who reported that the amount of calcium hydroxide consumed by the pozzolanic reaction is greater in high-fineness FA–cement pastes than in those with lower fineness.

**Figure 11:**
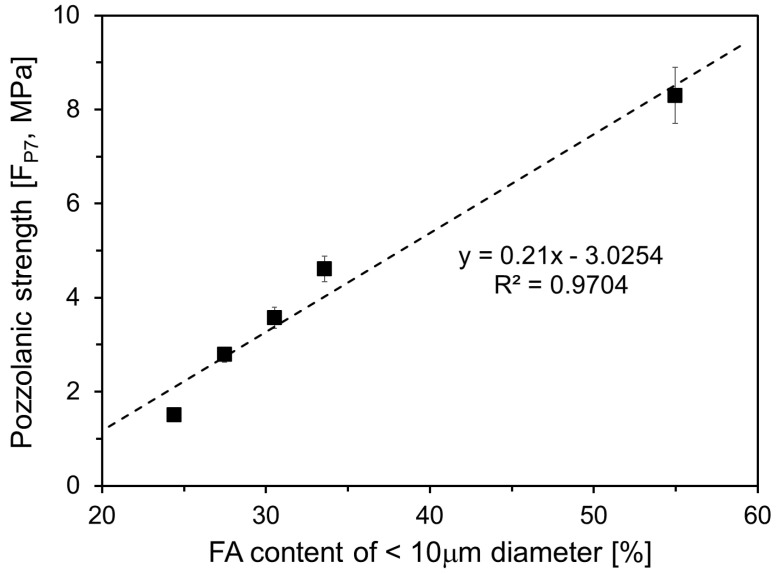
Pozzolanic strength obtained until 7 days.

**Figure 12:**
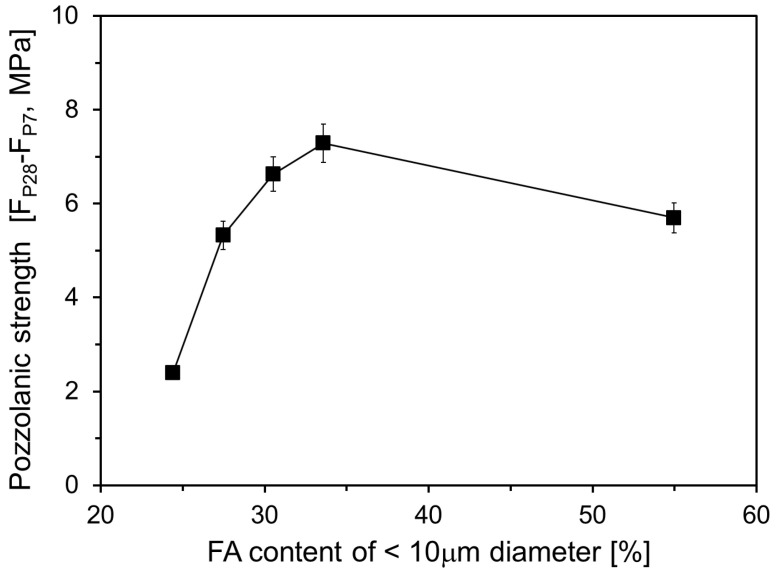
Pozzolanic strength obtained between 7 and 28 days.

The pozzolanic strength at different ages was calculated to assess the influence of FA particles smaller than 10 μm on the pozzolanic reaction. Figure 11 and Figure 12 show the pozzolanic strength for up to 7 d and from 7 d to 28 d, respectively. Figure 11 shows the increase in pozzolanic strength within the first 7 d, corresponding to the content of particles smaller than 10 μm in the FA, as presented in Table 2. The data indicate that as the proportion of particles smaller than 10 μm increases, pozzolanic strength also rises during the initial 7 d. With the standard-fineness FA, the pozzolanic strength increased by approximately 1.5 MPa, whereas using finer FA resulted in an increase of up to 8 MPa. The observed strength increase when using a finer FA can be explained by two mechanisms: first, a finer FA enhances the filling effect, contributing to greater compressive strength; smaller particles reduce the number of micropores in the cement paste, thereby increasing the overall compressive strength. Additionally, at early-age stages, a higher content of particles smaller than 10 μm likely promotes a more active pozzolanic reaction. Chindaprasirt et al. [34] found that finer FA has a higher glass content than coarser FA, indicating greater pozzolanic reactivity in finer FA. Thus, increasing the proportion of particles smaller than 10 μm in FA could be an effective strategy for improving the early-age strength of HVFA cement.

Figure 12 depicts the increase in pozzolanic strength between 7 d and 28 d relative to the proportion of particles smaller than 10 μm in the FA. The graph indicates that pozzolanic strength continues to increase as the content of particles smaller than 10 μm rises to 33.6%. However, the rate of increase slowed. When the content reached 55.0%, the pozzolanic strength decreased, likely owing to the higher content of fine particles accelerating the pozzolanic reaction, leading to greater FA consumption before 7 d. These results suggest that, over time, the differences in compressive strength related to the content of particles smaller than 10 μm may diminish.

**Figure 13:**
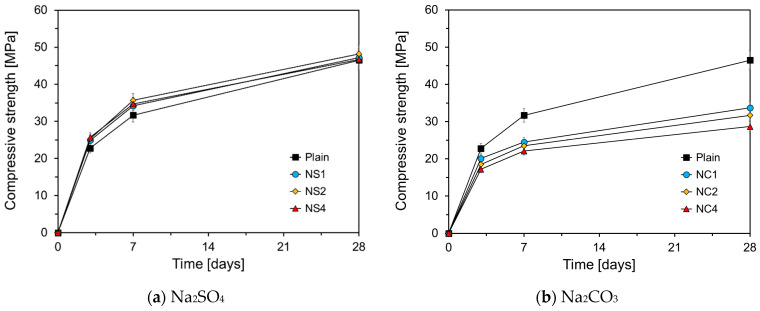

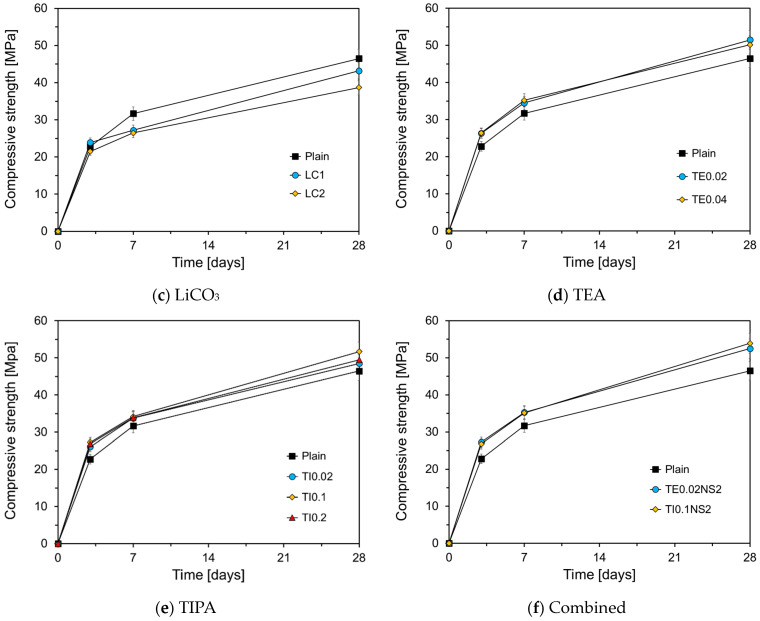
Effect of chemical agent on the compressive strength.

Figure 13 illustrates the effects of different chemical agents on compressive strength. Figure 13a shows the compressive strength results for the HVFA cement mortars with added Na_2_SO_4_. The results indicated that the specimens containing Na_2_SO_4_ exhibited compressive strengths 2–4 MPa higher than that of Plain at 3 d and 7 d, consistent with the increased specific heat release observed in Figure 3a as the Na_2_SO_4_ content increases. Additionally, the compressive strength at 3 d was largely unaffected by the amount of Na_2_SO_4_ added, consistent with the cumulative heat results in Figure 3b, which showed similar heat accumulation up to 72 h, regardless of the Na_2_SO_4_ content. However, no clear trend was observed regarding the influence of the Na_2_SO_4_ content. When Na_2_SO_4_ dissolves in water, Na^+^ and SO_4_^2−^ are generated. The dissolved Na^+^ generates NaOH, which increases the alkalinity of the solution, thus promoting the hydration of cement and the pozzolanic reaction of FA. SO_4_^2−^ reacts with calcium aluminate of OPC to precipitate Aft, thereby enhancing the early strength [35]. At 28 d, the compressive strength of the Na_2_SO_4_-treated specimens was only 0.2 to 1.7 Mpa higher than that of the Plain, indicating a reduced effect compared to earlier ages, suggesting that although Na_2_SO_4_ can enhance the early strength of HVFA cement mortar, its impact on long-term strength is limited.

Figure 13b shows the compressive strength results for specimens with added Na_2_CO_3_. The data revealed that increasing the Na_2_CO_3_ content led to a decrease in the compressive strength across all curing ages, contrary to the cumulative heat results shown in Figure 4. Although NC1 demonstrated a higher cumulative heat than Plain for up to 72 h (as shown in Figure 4b), its compressive strength at 3 d was significantly lower than that of the, as shown in Figure 13b. This discrepancy suggests that while Na_2_CO_3_ accelerates OPC hydration, it may alter the hydration products in a manner that ultimately reduces the strength. Janotk [36] reported that adding Na_2_CO_3_ to cement paste increases CaCO_3_ and decreases Ca(OH)_2_ levels. Additionally, although a higher Na_2_CO_3_ content may enhance the compressive strength on 1 d, it tends to reduce the strength on 28 d. As Na_2_CO_3_ reduces Ca(OH)_2_ in the long term, it may also inhibit the pozzolanic reaction of FA, making it unsuitable as a chemical activator to improve the compressive strength of HVFA cement mortar. Figure 13c shows the compressive strength results for the specimens with LiCO_3_. On 3 d, while LC1 showed a higher compressive strength than Plain, its compressive strength was lower on 7 d and 28 d. NC2 had a lower compressive strength than Plain at all ages. These results are consistent with the cumulative heat data in Figure 5b, where NC1 shows a cumulative heat similar to that of Plain for up to 72 h, whereas NC2 has a lower cumulative heat. When 2% LiCO3 was added, OPC hydration initially accelerated for up to 48 h but slowed thereafter.

Figure 13d shows the compressive strength results for the specimens with added TEA. The data reveal that TEA improved the compressive strength at all ages compared to Plain, which is consistent with the cumulative heat results in Figure 6b. Furthermore, the compressive strength of TE0.02 was higher than that of TE0.04 for all curing periods. TEA enhanced the dissolution of C_3_A in OPC, promoted ettringite formation [37,38], delayed the dissolution of C_3_S, and increased the formation of non-crystalline Ca(OH)_2_ [39]. As the TEA content increased, the available Ca(OH)_2_ for the pozzolanic reaction of FA decreased, likely leading to reduced compressive strength. Figure 13e shows the compressive strength results for the specimens with added TIPA. TIPA increased the compressive strength at all curing ages, with its effects being more pronounced at early ages. Although the impact of TIPA content was minimal up to 7 d, at 28 d, the compressive strength of TI0.1 was the highest. TIPA promoted the dissolution of Fe^3+^ and Al^3+^ ions from C_4_AF, thereby accelerating the silicate reaction during OPC hydration [40]. TIPA also consumes Ca(OH)_2_ to form Aft, which may inhibit the long-term pozzolanic reaction of the FA [41,42]. Figure 13f shows the compressive strength results for specimens with a combination of amine-based chemical activators and Na_2_SO_4._ At 3 d, the compressive strength of TI0.1, which used only TIPA, was higher than that of TE0.02NS2 and TI0.1NS2. However, at 7 d and 28 d, the compressive strengths of TE0.02NS2 and TI0.1NS2 were higher than those of TI0.1.

**Figure 14:**
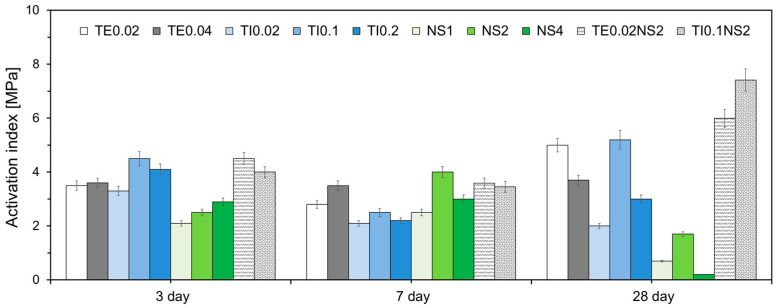
Activation index of specimens.

Figure 14 shows the activation indices of the specimens. The activation index was calculated as the difference between the compressive strengths of the specimens with chemical activators and Plain (Equation (2)).

Activation index = Strength of each specimen − Strength of Plain(2)

The NC and LC series were excluded from this analysis because their compressive strengths were lower than those of Plain. At 3 d, the specimens with TEA and TIPA showed a higher activation index than specimens with Na_2_SO_4_. TI series showed the highest activation index at 3 d. This shows that TIPA most significantly promotes hydration and the pozzolanic reaction of very early-age HVFA cement. On the other hand, at 7 d, the specimens with Na_2_SO_4_ exhibited the highest activation index. It can be seen that Na_2_SO_4_ promotes the reaction of HVFA cement the most after 3 days. At 28 d, the specimens with Na_2_SO_4_ exhibited the lowest activation index, whereas those with a combination of amine-based admixtures and Na_2_SO_4_ showed a higher activation index. In the case of HVFA cement, since the content of OPC is low, the Ca(OH)_2_ generated by the hydration of OPC is limited [43]. Therefore, even if the hydration of OPC and the pozzolanic reaction of FA are activated by Na_2_SO_4_, the effect is limited. Therefore, the strength enhancement effect seems to have decreased at 28 days.

## 4. Conclusions

This study investigated the effects of the FA particle size and chemical activators on the hydration and mechanical properties of HVFA cementitious materials. To prepare the HVFA cementitious specimens, 50% of the OPC was substituted with the same weight of FA. Five different types of FA were used, each varying in the proportion of particles smaller than 10 μm. In addition, chemical activators Na_2_SO_4_, Na_2_CO_3_, Li_2_CO_3_, TEA, and TIPA were employed. The specific heat flows and cumulative heat of the HVFA cement pastes were measured using isothermal calorimetry. The compressive strengths of the HVFA mortars were evaluated according to ISO 679 at 3 d, 7 d, and 28 d. The results of this study can be summarized as follows.

1. The FA particle size significantly affects the early-age hydration of HVFA cement pastes. An increase in the contents of FA particles with a diameter lower than 10 μm led to higher specific heat flow, indicating that more OPC undergoes hydration due to the filling effect of FA particles.
2. The early-age hydration of HVFA cement pastes varied depending on the type of chemical activator used. Na_2_SO_4_, TEA, and TIPA consistently promoted early hydration, regardless of their concentration. In contrast, Na_2_CO_3_ promoted or delayed hydration depending on its concentration. Li_2_CO_3_ enhanced the hydration of HVFA cement pastes for up to 48 h but delayed it thereafter. The early-age hydration of HVFA cement pastes was significantly promoted when Na_2_SO_4_ was combined with TEA or TIPA.
3. The compressive strength of HVFA mortar increased with a higher proportion of FA particles smaller than 10 μm. Specifically, the compressive strength of FH-50 was approximately 30%, 20%, and 20% greater than that of Plain at 3 d, 7 d, and 28 d, respectively. The pozzolanic strength increased linearly until 7 d, correlating with the content of FA particles smaller than 10 μm. Between 7 d and 28 d, the pozzolanic strength increased continuously as the fine particle content increased to 33.6%. However, the pozzolanic strength decreased when the filler content reached 55.0%.
4. The compressive strength of the HVFA mortars differed depending on the type of chemical activator used. Na_2_SO_4_, TEA, and TIPA enhanced the compressive strength of the HVFA mortars regardless of their concentrations, whereas Na_2_CO_3_ and Li_2_CO_3_ reduced the compressive strength of the HVFA mortar. The highest compressive strength was achieved in the HVFA mortar using Na_2_SO_4_ combined with amine-based activators such as TEA and TIPA.

## Figures and Tables

**Table 1:** Chemical compositions of OPC and FA.

	Chemical Compositions (wt. %)								
	CaO	SiO_2_	Al_2_O_3_	Fe_2_O_3_	MgO	K_2_O	Na_2_O	SO_3_	LOI
OPC	62.3	20.8	4.3	3.1	4.4	0.6	0.3	1.5	1.3
FA	3.9	58.7	21.8	6.7	1.8	1.2	1.1	0.4	2.2

**Table 2:** Proportion of FA with a particle size of 10 μm or less.

	Content (wt.%)				
	FN	0.9FN + 0.1FH	0.8FN + 0.2FH	0.7FN + 0.3FH	FH
<10 μm	24.4	27.5	30.5	33.6	55.0

**Table 3:** Mixing proportions of FA cement pastes.

	Binder (wt. %)			Activator (wt.% by Binder)				
	OPC	FN	FH	TEA	TIPA	Na_2_SO_4_	Na_2_CO_3_	LiCO_3_
Plain	50	50	-	-	-	-	-	-
Plain-Q *	50	50 *	-	-	-	-	-	-
FH-10	50	45	5	-	-	-	-	-
FH-20	50	40	10	-	-	-	-	-
FH-30	50	35	15	-	-	-	-	-
FH-50	50	-	50	-	-	-	-	-
TE0.02	50	50	-	0.02	-	-	-	-
TE0.04	50	50	-	0.04	-	-	-	-
TI0.02	50	50	-	-	0.02	-	-	-
TI0.1	50	50	-		0.1	-	-	-
TI0.2	50	50	-	-	0.2	-	-	-
NS1	50	50	-	-	-	1	-	-
NS2	50	50	-	-	-	2	-	-
NS4	50	50	-	-	-	4	-	-
NC1	50	50	-	-	-	-	1	-
NC2	50	50	-	-	-	-	2	-
NC4	50	50	-	-	-	-	4	-
LC1	50	50	-	-	-	-	-	1
LC2	50	50	-	-	-	-	-	2
TE0.02NS2	50	50	-	0.02	-	2	-	-
TI0.1NS2	50	50	-	-	0.1	2	-	-

* An inert quartz powder with a PSD similar to that of FN was used as a substitute for FN.

## Data Availability

The original contributions presented in the study are included in the article, further inquiries can be directed to the corresponding author.
